# Three new species of the genus *Coddingtonia* from Asia (Araneae, Theridiosomatidae)

**DOI:** 10.3897/zookeys.886.35492

**Published:** 2019-11-05

**Authors:** Chengcheng Feng, Yucheng Lin

**Affiliations:** 1 Key Laboratory of Bio-resources and Eco-environment (Ministry of Education), College of Life Sciences, Sichuan University, Chengdu, Sichuan 610064, China Sichuan University Chengdu China; 2 The Sichuan Key Laboratory for Conservation Biology of Endangered Wildlife, Sichuan University, Chengdu, Sichuan 610064, China Sichuan University Chengdu China

**Keywords:** China, Indonesia, new genus record, new species, ray spider, taxonomy

## Abstract

The current paper expands knowledge of the genus *Coddingtonia* Miller, Griswold & Yin, 2009. Based on morphological characters and molecular data, three species are documented as new to science: *C. erhuan* Feng & Lin, **sp. nov.** (♀) from China, *C. lizu* Feng & Lin, **sp. nov.** (♀) from China, and *C. huifengi* Feng & Lin, **sp. nov.** (♂♀) from Indonesia. The type of *C. euryopoides* Miller, Griswold & Yin, 2009 is also reexamined. DNA sequences (COI), detailed illustrations of habitus, male palp and epigyne are provided for these four species, as well as a key and a distribution map for *Coddingtonia* species.

## Introduction

*Coddingtonia* was originally established by Miller et al. (2009) as a monotypic genus based on *C. euryopoides* Miller et al., 2009 from the Gaoligong Mountains in Southwest China. Labarque and Griswold (2014) reported two *Coddingtonia* species from Laos and Malaysia. Currently the genus *Coddingtonia* contains three valid species distributed in China, Laos, Thailand, and Malaysia (Lopardo and Hormiga 2015; World Spider Catalog 2019).

In a recent collection of theridiosomatids from China and Indonesia, we found three members of *Coddingtonia* and propose them as species new to science. Detailed diagnoses, descriptions, and identifying illustrations are provided for each. This work also represents the first record of this genus from Indonesia.

## Materials and methods

All specimens were preserved in 95% ethanol. Specimens were examined and measured with a Leica M205 C stereomicroscope. Further details were studied using an Olympus BX53 compound microscope mounted with a Canon EOS 60D wide zoom digital camera (8.5 megapixels). Male and female copulatory organs were examined and photographed after they were dissected and detached from the bodies. Vulvae were treated with lactic acid before being photographed. The digital images were montaged using Helicon Focus 3.10 image stacking software (Khmelik et al. 2006). All measurements in the paper are in millimeters. Leg measurements are given in the following sequence: total length (femur, patella, tibia, metatarsus, and tarsus).

Abbreviations in figures are as follows:

**C** conductor;

**CD** copulatory ducts;

**CP** central pit;

**CY** cymbium;

**E** embolus;

**EA** mesial embolic apophysis;

**FD** fertilization ducts;

**GD** glandular ducts;

**LG** lateral grooves;

**LW** lateral wing;

**MA** median apophysis;

**S** spermathecae;

**ST** subtegulum;

**T** tegulum.

A partial fragment (636 bp) of the mitochondrial gene cytochrome *c* oxidase subunit I (COI) was amplified and sequenced in order to check the genetic distance between morphologically close related species and confirm identifications and the sex pairing accuracy. For the same reasons, sequences of *Coddingtonia euryopoides* Miller et al., 2009 were also included.

The primers used are as following: LCO1490 (5’-GGTCAACAAATCATCATAAAGATATTGG-3’) and HCO2198 (5’-TAAACTTCAGGGTGACCAAAAAA TCA-3’). Raw sequences were edited and assembled using BioEdit v.7.2.5 (Hall 1999) and the uncorrected pairwise distance between the species was calculated using MEGA7.0.14 (Kumar et al. 2016). All sequences were incorporated in GenBank and the accession numbers are provided in Table 1. Results of the comparison between the genetic distances are shown in Table 2.

**Table 1.** Voucher specimen information.

Species	Sample	GenBank accession number	Geographical coordinates
*C. erhuan* sp. nov.	1♂ juv.	MN211319	27°08.28'N, 098°49.34'E
	1♀	MN211318	
*C. euryopoides*	1♂ juv.	MN211317	24°49.73'N, 098°45.60'E
	1♀	MN211316	
*C. lizu* sp. nov.	1♂ juv.	MN211313	18°35.86'N, 109°25.61'E
	1♀	MN211312	
*C. huifengi* sp. nov.	1♂	MN211315	00°15.74'S, 100°18.49'E
	1♀	MN211314	

**Table 2.** Uncorrected genetic pairwise distance (lower triangle) and standard errors (upper triangle) of the COI partial sequence between species discussed in the text.

	Species		1		2		3		4	
			♀	♂ juv.	♀	♂ juv.	♀	♂ juv.	♀	♂
1	*C. erhuan* sp. nov.	♀		0.000	0.014	0.014	0.015	0.015	0.015	0.015
		♂ juv.	0.000		0.014	0.014	0.015	0.015	0.015	0.015
2	*C. euryopoides*	♀	0.135	0.135		0.000	0.016	0.016	0.016	0.016
		♂ juv.	0.135	0.135	0.000		0.016	0.016	0.016	0.016
3	*C. lizu* sp. nov.	♀	0.139	0.139	0.152	0.152		0.000	0.016	0.016
		♂ juv.	0.139	0.139	0.152	0.152	0.000		0.016	0.016
4	*C. huifengi* sp. nov.	♀	0.137	0.137	0.140	0.140	0.150	0.150		0.000
		♂	0.137	0.137	0.140	0.140	0.150	0.150	0.000	

All examined materials are deposited in the Natural History Museum of Sichuan University in Chengdu (**NHMSU**), China, except the holotype of *C. euryopoides*, which is deposited in the School of Life Sciences, Hunan Normal University in Changsha (**HNU**), China.

## Taxonomy

### Family Theridiosomatidae Simon, 1881

#### Genus Coddingtonia

Taxon classificationAnimaliaAraneaeTheridiosomatidae

Miller et al., 2009

771B619E-6A4E-5E1E-84AC-567B9C728D48

Coddingtonia Miller, Griswold & Yin, 2009: 30.
Luangnam Wunderlich, 2011: 431.
Coddingtonia : Labarque and Griswold 2014: 419 (synonymized with Luangnam).

##### Type species.

*Coddingtonia euryopoides* Miller et al., 2009 by original designation.

##### Diagnosis.

**Figure 1.**
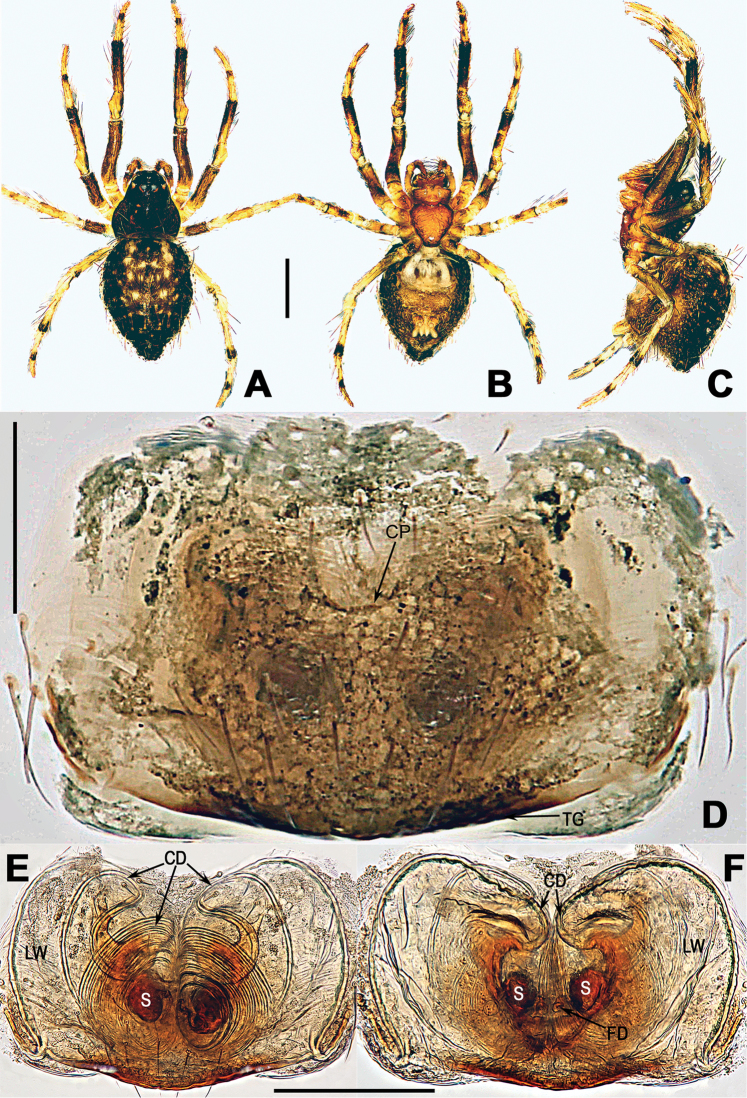
*Coddingtonia euryopoides* Miller et al., 2009, holotype female. **A–C** Habitus **D** epigyne **E, F** vulva (lactic acid-treated) **A, F** dorsal **B, D, E** ventral **C** lateral. Abbreviations: CD copulatory ducts; CP central pit; FD fertilization ducts; GD glandular ducts; LW lateral wings; S spermathecae. Scale bars: 0.50 mm (**A, C**); 0.20 mm (**D–F**).

**Figure 2.**
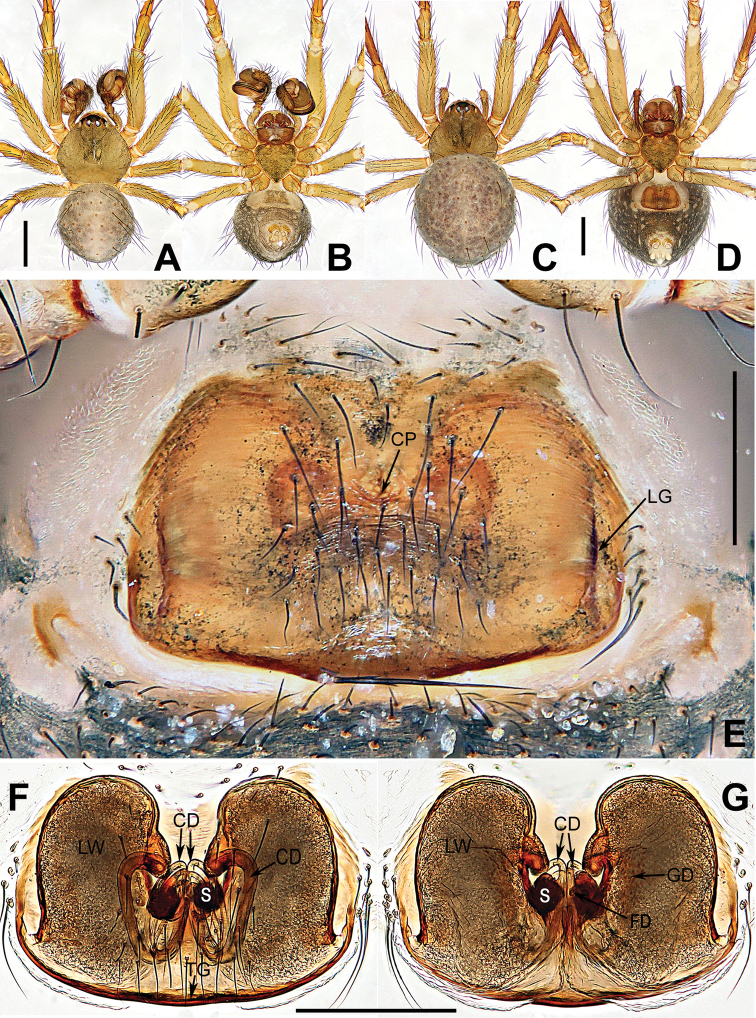
*Coddingtonia huifengi* sp. nov., holotype male (**A, B**) and paratype female (**C–G**). **A–D** Habitus **E** epigyne **F, G** vulva (lactic acid-treated). **A, C, G** dorsal **B, D–F** ventral. Abbreviations: CD copulatory ducts; CP central pit; FD fertilization ducts; GD glandular ducts; LG lateral grooves; LW lateral Wings; TG Transversal groove; S spermathecae. Scale bars: 0.50 mm (**A–D**); 0.20 mm (**E–G**).

**Figure 3.**
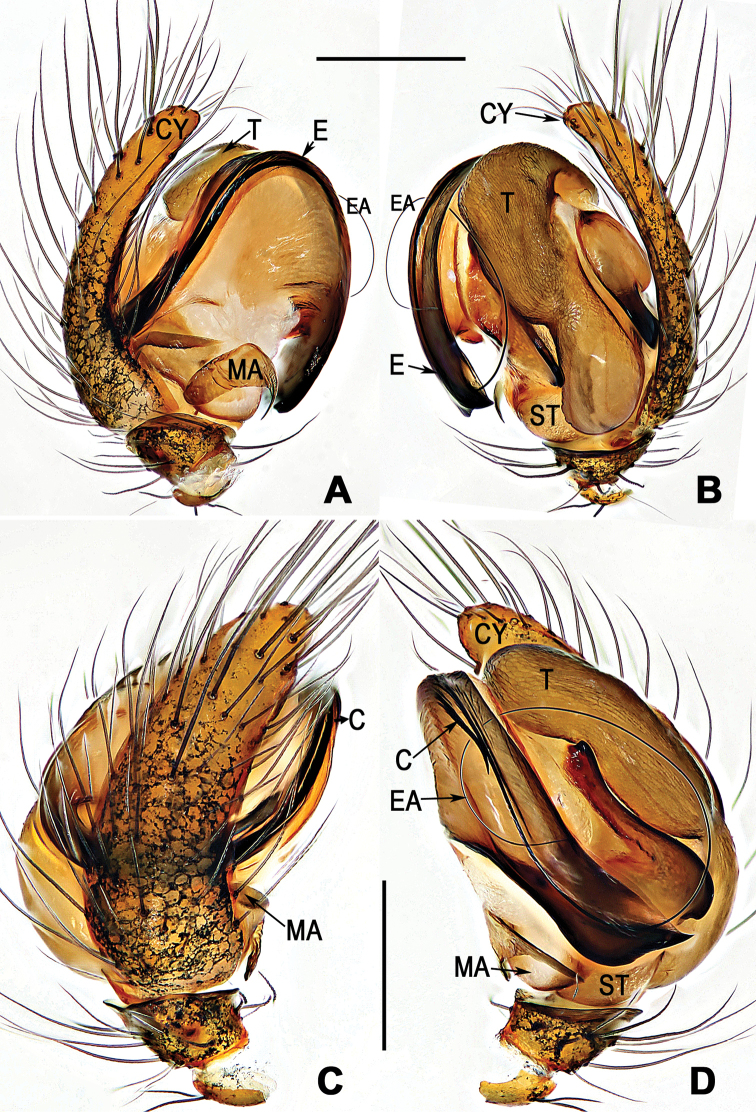
*Coddingtonia huifengi* sp. nov., holotype male. **A–D** Left palp **A** prolateral **B** retrolateral **C** dorsal **D** ventral. Abbreviations: C conductor; E embolus; T tegulum; CY cymbium; EA embolic apophysis; MA median apophysis; ST subtegulum. Scale bars: 0.20 mm.

**Figure 4.**
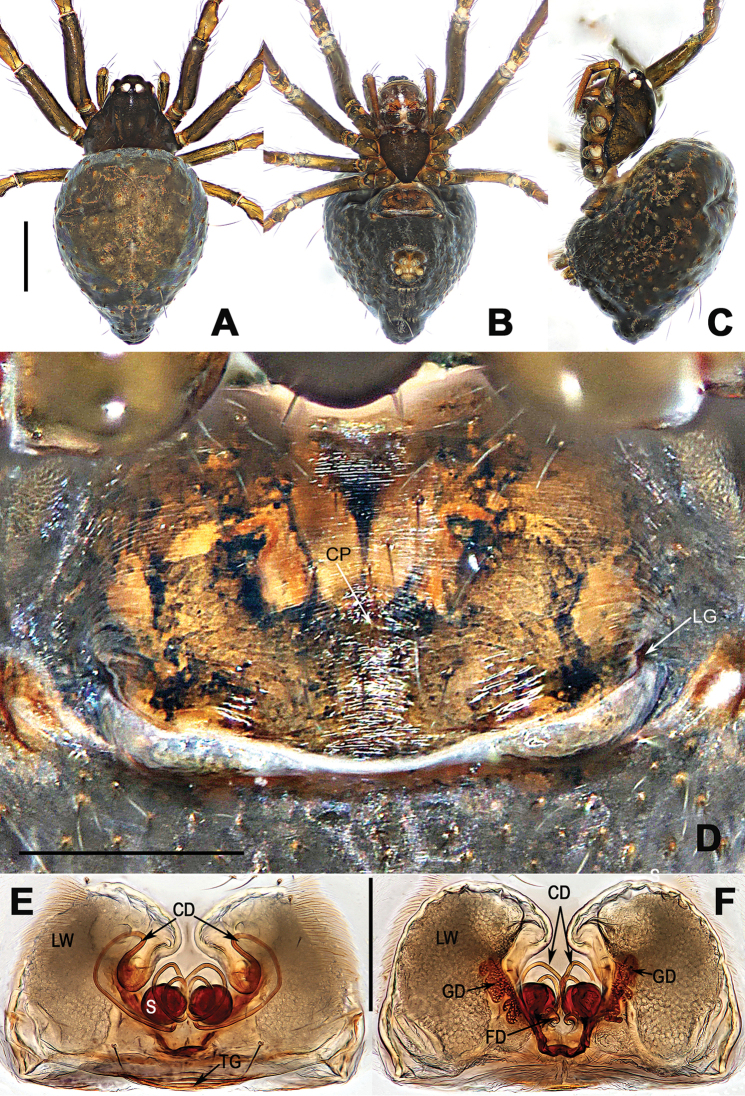
*Coddingtonia erhuan* sp. nov., holotype female. **A–C** Habitus **D** epigyne **E, F** vulva (lactic acid-treated) **A, F** dorsal **B, D, E** ventral **C** lateral. Abbreviations: CD copulatory ducts; CP central pit; FD fertilization ducts; GD glandular ducts; LG lateral grooves; LW lateral wings; TG transversal groove; S spermathecae. Scale bars: 0.50 mm (**A–C**); 0.20 mm (**D–F**).

**Figure 5.**
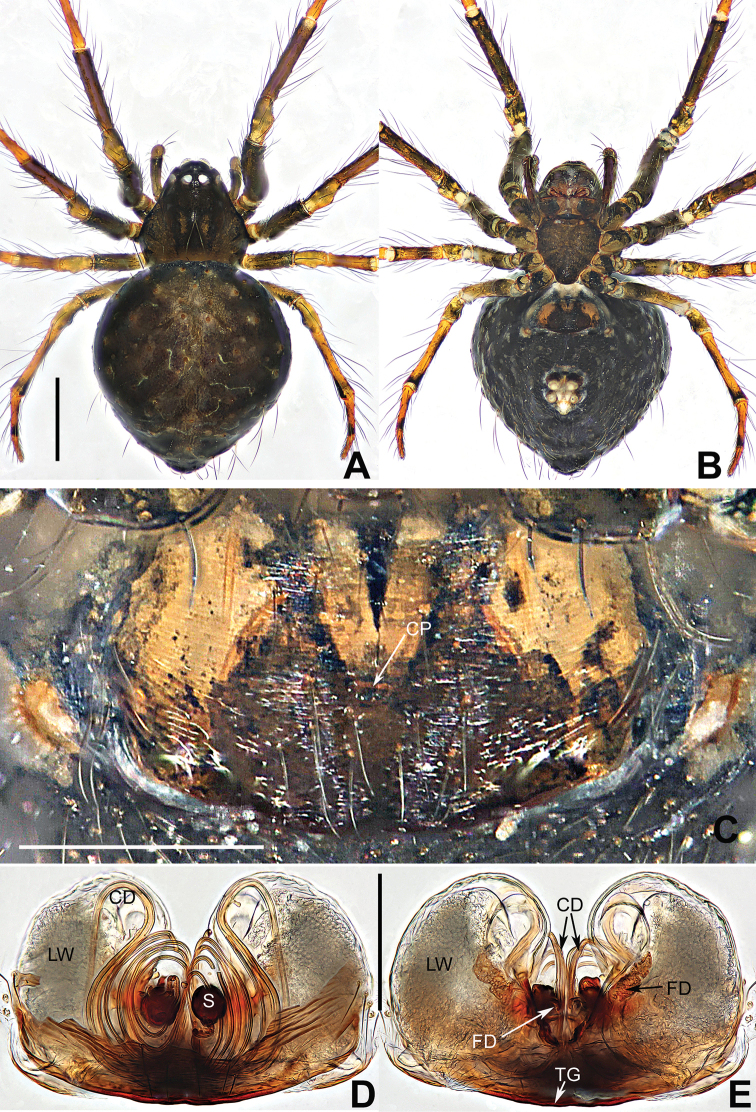
*Coddingtonia lizu* sp. nov., holotype female. **A, B** Habitus **C** epigyne **D, E** vulva (lactic acid-treated) **A, E** dorsal **B–D** ventral. Abbreviations: CD copulatory ducts; CP central pit; FD fertilization ducts; GD glandular ducts; LW lateral wings; S spermathecae. Scale bars 0.50 mm (**A, B**); 0.20 mm (**C–E**).

The male of *Coddingtonia* may be distinguished from other theridiosomatids by the mesal bristle of the embolic apophysis (Fig. 3A, B, D; Wunderlich 2011: figs 3, 5). The female of *Coddingtonia* can be distinguished from other theridiosomatids by the following combination of characters: spermathecae separated by about one diameter (Figs 1E, F, 2F, G, 4E, F, 5D, E) vs. juxtaposed and partially fused together (Coddington, 1986), long and coiled copulatory ducts surrounding the spermathecae, but lacking that in other theridiosomatids (Figs 1E, F, 2F, G, 4E, F, 5D, E).

##### Composition.

*Coddingtonia anaktakun* Labarque & Griswold, 2014 (Malaysia), *C. erhuan* sp. nov. (China), *C. discobulbus* (Wunderlich, 2011) (Laos), *C. euryopoides*Miller et al. 2009 (China), *C. huifengi* sp. nov. (Indonesia), and *C. lizu* sp. nov. (China).

##### Distribution.

**Figure 6.**
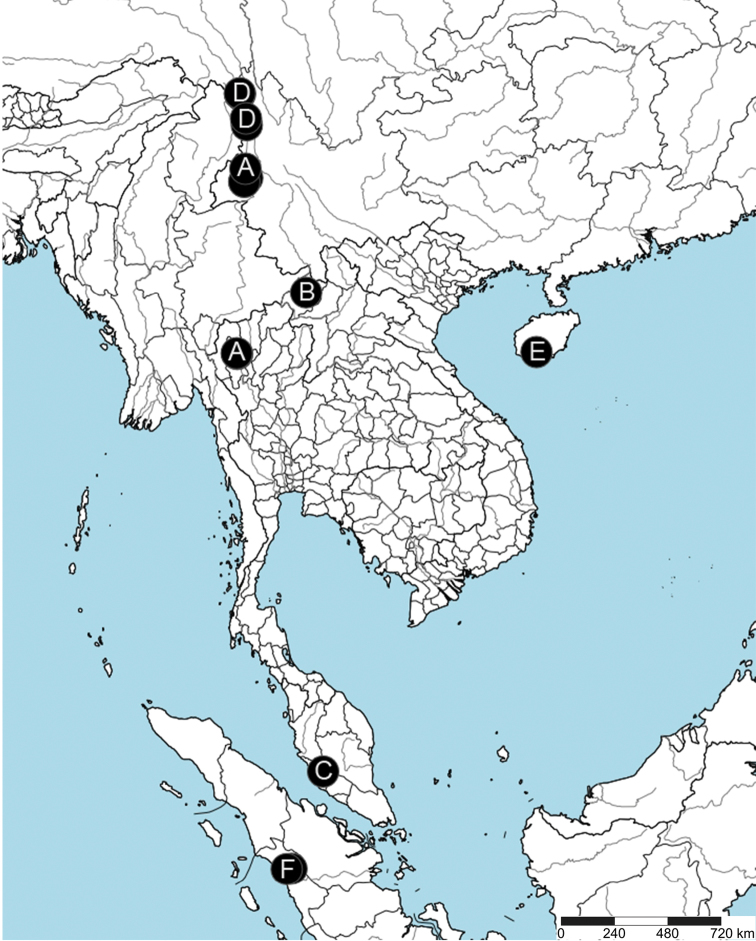
Distribution records of *Coddingtonia* spp. in the world. **A***C. euryopoides* Miller et al., 2009 **B***C. discobulbus* Wunderlich, 2011 **C***C. anaktakun* Labarque & Griswold, 2014 **D***C. erhuan* sp. nov. **E***C. lizu* sp. nov. **F***C. huifengi* sp. nov.

Southern China (Yunnan, Hainan), Laos, Thailand, Malaysia, and Indonesia (Fig. 6).

### Key to species of *Coddingtonia*

(Only referring to characters of the vulva)

**Table:** 

1	Spermatheca round (Figs 1D, E, 2F, G, 4E, F, 5D, E)	**2**
–	Spermatheca oval (Labarque and Griswold 2014: fig. 7E, F)	***C. discobulbus***
2	Copulatory duct forms 2 coils (Fig. 4E, F)	***C. erhuan* sp. nov.**
–	Copulatory duct with more than 2 coils (Figs 1E, F, 2F, G, 5D, E)	**3**
3	Copulatory duct with 3 coils (Fig. 2F, G)	***C. huifengi* sp. nov.**
–	Copulatory duct with more than 3 coils (Figs 1D, E, 5D, E)	**4**
4	Copulatory duct with 5 coils (Fig. 5D, E)	***C. lizu* sp. nov.**
–	Copulatory duct with 6 or 9 coils (Fig. 1D, E)	**5**
5	Copulatory duct with 9 coils (Fig. 1E, F)	***C. euryopoides***
–	Copulatory duct with 6 coils (Labarque and Griswold 2014: fig. 6E, F)	***C. anaktakun***

#### Coddingtonia euryopoides

Taxon classificationAnimaliaAraneaeTheridiosomatidae

Miller et al., 2009

3EF87468-32F4-54AE-B842-412082426632

Fig. 1

Coddingtonia euryopoides Miller et al., 2009: 30, figs 8B, 11E, F (♀); Lopardo and Hormiga 2015: 734, figs 124A–F, 125A–G, 137D (♀).

##### Material examined.

***Holotype*** ♀ (CASENT 9022403 in HNU) CHINA: Yunnan Province, Longling County, Mangkuan Township, Zaotang He at Baihualing Village, 25°18.27'N, 98°48.04'E, ca. 1635 m, 2 Jun. 2005, good subtropical broadleaf forest, dusting webs in understory, C. Griswold leg.

##### Other material examined.

1♀, 3♂ juv. (NHMSU) CHINA: Yunnan Province: Longling County, Longjiang Town, Xiaoheishan Nature Reserve, 24°49.73'N, 98°45.60'E, ca. 2020 m, 22 Aug. 2018, Y. Lin et al. leg. Of them, 1♂ juv. and 1♀ used for sequencing, same data for preceding, GenBank: MN211317 and MN211316; 1♀ (NHMSU): Baoshan City, Tengchong County, Gudong Town, Jiangdong Village, Jiangdong Hill, Luoshui Cave, 24°58.10'N, 98°52.10'E, ca. 1880 m, 16 Nov. 2013, Y. Li & J. Liu leg.

##### Diagnosis.

The male of *C. euryopoides* differs from the males of other species by the mesal bristle of the embolic apophysis describing a semi-loop very close to the embolus base and a semi-loop around the bulb, and the straight median apophysis having a tapering tip (Lopardo and Hormiga 2015: fig. 124B, E, F). The female of *C. euryopoides* can be distinguished from the other five species by having 9 coils of the copulatory ducts (Fig. 1E), whereas other species have fewer coils. Moreover, *C. euryopoides* differs from *C. anaktakun*, *C. discobulbus*, and *C. huifengi* sp. nov. by having a posterior tubercle on the abdomen (Fig. 1A–C), whereas this tubercle is absent in the latter three species.

##### Description.

See Fig. 1A–F and Miller et al. (2009: 30). Male of this species remains unknown.

##### Distribution.

China (Yunnan) and Thailand (Chiang Mai) (Fig. 6).

#### Coddingtonia huifengi sp. nov.

Taxon classificationAnimaliaAraneaeTheridiosomatidae

E6125CDF-52BB-5D82-A8FA-044F313DFD4B

http://zoobank.org/2E2BF8FD-526A-4CBF-9EC6-F5E0A7BD64DE

Figs 2 , 3

##### Type material.

***Holotype*** ♀, ***paratypes*** 2♂ and 28♀ (NHMSU) INDONESIA: Kanagarian Matuailia, environs of Batang Lawang Cave, 0°15.74'S, 100°18.49'E, ca. 760 m, 12 Jan. 2014, H. Zhao leg. Two paratypes 1♂ and 1♀ used for sequencing, same data as for preceding, GenBank: MN211315 and MN211314; 1♂, 2♀ (NHMSU) Sumatra, West Sumatra Province, Kab Agam TaBik Simarasok Village, Jorong Koto tuo, 0°14.90'S, 100°28.99'E, ca. 710 m, 11 Jan. 2014, H. Zhao leg.

##### Etymology.

The new species is named after Dr Huifeng Zhao who extensively collected spiders from Southeast Asia.

##### Diagnosis.

The male of this new species differs from the male of *C. euryopoides* by the median apophysis with a distal flexible hook, and the narrower, shorter conductor (Fig. 3A, D); in other similar species the tip is straight and wider and conductor is longer (see Labarque and Griswold 2014: figs 1C, 5D–F). The female can be distinguished from the other five species by having 3 coils (one thick, two thin) of copulatory ducts (Fig. 2F), whereas they are fewer or more in other species. Moreover, *C. huifengi* differs by the lack of a posterior tubercle on the abdomen (Fig. 2A–D) vs. present in *C. euryopoides*, *C. erhuan* sp. nov., and *C. lizu* sp. nov. (Figs 1A–C, 4A–C, 5A, B).

##### Description.

**Females** (holotype). Carapace nearly pentagonal, dim yellowish, cephalic area moderately raised. Anterior eye row precurved, posterior eye row straight. Sternum heart-shaped, grey yellow, with sparse setae. Mouthparts brown. Femora and patellae dim yellow, other segments brown. Abdomen round, dorsally grey, ventrally deeper, bears sparse long hairs, weakly ossified at hair base (Fig. 2A, B). *Measurements*: total length 2.13. Carapace 1.02 long, 0.97 wide. Clypeus 0.15 high. Sternum 0.48 long, 0.46 wide. Abdomen 1.41 long, 1.35 wide. Length of legs: I 2.78 (0.85, 0.30, 0.73, 0.50, 0.40); II 2.66 (0.84, 0.23, 0.71, 0.47, 0.41); III 1.79 (0.56, 0.16, 0.45, 0.35, 0.27); IV 2.35 (0.79, 0.21, 0.58, 0.43, 0.34).

*Epigyne* (Fig. 2E–G): epigyne covered with sparse black setae in the central region; with deep central pit and 2 longitudinal grooves close to lateral margins of the plate. Spermathecae barely visible through the integument; LW well developed, like a pair of boxing gloves, swollen sacks with dorso-median glandular ducts; spermathecae globular, separated by one radius; copulatory ducts form an expanded posterolateral loop, and coiled into 2 slender posteromedian loops, finally connecting ventrally on the spermathecae; fertilization ducts arise from the dorsomesal the spermathecae.

**Male** (one paratype): Somatic features as in Fig. 2A, B and coloration slightly darker than in female. *Measurements*: Total length 1.87. Carapace 0.98 long, 0.93 wide. Clypeus 0.16 high. Sternum 0.46 long, 0.45 wide. Abdomen 0.92 long, 0.89 wide. Length of legs: I 2.33 (0.73, 0.24, 0.61, 0.40, 0.35); II 2.09 (0.66, 0.19, 0.53, 0.39, 0.32); III 1.54 (0.48, 0.15, 0.35, 0.30, 0.26); IV 1.90 (0.61, 0.20, 0.45, 0.36, 0.28).

*Palp* (Fig. 3A–D): tibia small, cymbium narrow, about 2 times longer than width, with long setae; paracymbium short and small, about of 1/5 cymbial length; tegulum capacious; median apophysis lamellar, subrectangular; conductor disk shaped with a needle-like distal process; mesal bristle of the embolic apophysis describes a semi-loop above the tegulum and cymbium; embolus long, whip-like, extending far beyond the mesial embolic apophysis and coiling into one loop.

##### Distribution.

Known only from the type locality (Fig. 6).

#### Coddingtonia erhuan sp. nov.

Taxon classificationAnimaliaAraneaeTheridiosomatidae

4CD682FE-F17C-5276-A96F-EDC795361848

http://zoobank.org/15CE2D97-4B6B-4CD7-B117-CE15B9FF1A1E

Fig. 4

##### Type material.

***Holotype*** ♀, ***paratypes*** 5♀ and 1♂ juv. (NHMSU) CHINA: Yunnan Province, Gaoligongshan, the west of Nujiang River, Shibali Village, 27°08.28'N, 98°49.34'E, ca. 1850 m, 19 Aug. 2018, Y. Lin et al. leg.; Two paratypes 1♂ juv. and 1♀ used for sequencing, same data as preceding, GenBank: MN211319 and MN211318.

##### Other material examined.

2♀ (NHMSU) CHINA: Yunnan Province, Gongshan County, Sijitong Village, on the banks of Nujiang River, 8°03.27'N, 98°35.76'E, ca. 1620 m, 12 Aug. 2018; 1♀ (NHMSU) CHINA: Yunnan Province, Longling County, Mangkuan Town, Baihualing Village, Zaotang River, subtropical broadleaf forest, 25°18.27'N, 98°48.04'E, ca. 1640 m, 21 Aug. 2018; 2♀ (NHMSU) CHINA: Yunnan Province, Longling County, Longjiang Town, Xiaoheishan Nature Reserve, Gucheng Hill, broadleaved deciduous forest, in surface leaf litter, 24°49.73'N, 98°45.55'E, ca. 2010 m, 22 Aug. 2018; 2♀ (NHMSU) CHINA: Yunnan Province, Gongshan County, the road of from Bingzhongluo Town to Puhuasi Temple, broadleaved deciduous forest litter, 28°01.42'N, 98°36.13'E, ca. 1870 m, 12 Aug. 2018, Y. Lin et al. leg.; 1♀ (NHMSU) CHINA: Yunnan Province, Fugong County, Shangpa Village, broadleaves deciduous forest, 26°53.66'N, 98°51.16'E, ca. 1470 m, 2 Jul. 2016, Y. Li leg.

##### Etymology.

Formed from the Chinese words for two (èr 二) and circle (huán 环), referring to the paired loops of copulatory ducts (Fig. 4E); noun.

##### Diagnosis.

This new species can be distinguished from other congeners by the 2 coils of the unilateral copulatory ducts around the spermathecae (Fig. 4E–F).By having a posterior tubercle on the abdomen (Fig. 4A–C) it differs from *C. huifengi* sp. nov. (Fig. 2A–D), *C. anaktakun* and *C. discobulbus* (Labarque and Griswold 2014: figs 5A–C, 6A–C, 7A–C).

##### Description.

**Female** (holotype): Carapace pear-shaped, black. Sternum tan. Legs dark brown. Abdomen obovate with posterior tubercle, dark black, ventrally darker than dorsally, covers sparse setae (Fig. 4A–C). *Measurements*: Total length 1.66. Carapace 0.62 long, 0.60 wide. Clypeus 0.14 high. Sternum 0.37 long, 0.38 wide. Abdomen 1.21 long, 1.04 wide. Length of legs: I 2.03 (0.65, 0.25, 0.43, 0.39, 0.31); II 1.80 (0.55, 0.23, 0.38, 0.34, 0.30); III 1.39 (0.42, 0.18, 0.26, 0.28, 0.25); IV 1.78 (0.55, 0.23, 0.40, 0.34, 0.26).

*Epigyne* (Fig. 4D–F): plate weakly sclerotized, nearly rectangular, with an indistinct central pit and pair of posterolateral pockets. Spermathecae barely visible through the integument; lateral wings well developed, with sclerotized glandular ducts in the dorso-medial; spermathecae globose, closely spaced and almost adjacent; copulatory ducts form a half loop in the ventral later wings, followed by 2 complete loops surround the spermathecae, and finally connect to the spermathecae (Fig. 4E); fertilization ducts short and twisty, arise from the dorsal side of spermathecae (Fig. 4F).

**Male**. unknown.

##### Distribution.

Known only from the type locality (Fig. 6).

#### Coddingtonia lizu sp. nov.

Taxon classificationAnimaliaAraneaeTheridiosomatidae

25D592E8-AA13-52D3-864E-907E039E9A8C

http://zoobank.org/4DBF94AC-E4F0-4937-BD47-FB9709598596

Fig. 5

##### Type material.

***Holotype*** ♀, ***paratypes*** 2♀ and 2 juv. ♂ (NHMSU) CHINA: Hainan Province, Sanya City, Baoting County, Maogan Town, Xian’an Stone Cave, 18°35.86'N, 109°25.61'E, ca. 620 m, 24 Nov. 2014, F. Li et al. leg. Two paratypes 1 juv. ♂ and 1♀ used for sequencing, same data as for preceding, GenBank: MN211313 and MN211312.

##### Etymology.

Named for the Lizu people, an ethnic minority that first settled in the Hainan Province. Noun in apposition.

##### Diagnosis.

This new species can be distinguished from the congeners by having 5 loops of unilateral copulatory duct (Fig. 5D; Note: the broken first and fourth loops on the right side of copulatory duct in vulva are due to careless dissection). Moreover, it has a posterior tubercle on the abdomen (Fig. 5A, B), which is absent in *C. anaktakun*, *C. discobulbus*, and *C. huifengi* sp. nov. (Fig. 2A–D; Labarque and Griswold 2014: figs 5A–C, 6A–C, 7A–C).

##### Description.

**Female** (holotype): Carapace pear-shaped, black. Sternum dim, posteriorly contracted. Femora and tibiae of legs dark, other segments yellow to brown. Abdomen dark black, dorsal color lighter than venter, with posterior tubercle, covers sparse long, stiff setae (Fig. 5A, B). *Measurements*: Total length 1.72. Carapace 0.64 long, 0.61 wide. Clypeus 0.13 high. Sternum 0.40 long, 0.38 wide. Abdomen 1.23 long, 1.05 wide. Length of legs: I 2.06 (0.66, 0.26, 0.43, 0.39, 0.32); II 1.84 (0.56, 0.23, 0.38, 0.35, 0.32); III 1.42 (0.43, 0.19, 0.26, 0.29, 0.25); IV 1.81 (0.56, 0.23, 0.41, 0.34, 0.27).

*Epigyne* (Fig. 5C–E): weakly sclerotized, nearly rectangular, black pigmentation in the central region; central pit and lateral pockets indistinct. lateral wings well developed, reniform and translucent; spermathecae small and round, separated by approximately one radius; copulatory ducts form a posterolateral auricular loop on the both sides of the lateral wings, followed by 5 loops, and finally connecting ventrally on the spermathecae (Fig. 5D); fertilization ducts short, arise from the dorsal-inner base of spermathecae (Fig. 5E).

**Male**. unknown.

##### Distribution.

Known only from the type locality (Fig. 6).

## Supplementary Material

XML Treatment for Coddingtonia

XML Treatment for Coddingtonia euryopoides

XML Treatment for Coddingtonia huifengi

XML Treatment for Coddingtonia erhuan

XML Treatment for Coddingtonia lizu
